# HGCPep: Hypergraph Deep Learning Identifies Cancer-associated Non-coding Peptides

**DOI:** 10.1093/gpbjnl/qzaf093

**Published:** 2025-12-02

**Authors:** Wentao Long, Zhongshen Li, Junru Jin, Jianbo Qiao, Yu Wang, Leyi Wei

**Affiliations:** School of Software, Shandong University, Jinan 250101, China; Joint SDU-NTU Centre for Artificial Intelligence Research (C-FAIR), Shandong University, Jinan 250101, China; School of Software, Shandong University, Jinan 250101, China; Joint SDU-NTU Centre for Artificial Intelligence Research (C-FAIR), Shandong University, Jinan 250101, China; School of Software, Shandong University, Jinan 250101, China; Joint SDU-NTU Centre for Artificial Intelligence Research (C-FAIR), Shandong University, Jinan 250101, China; School of Software, Shandong University, Jinan 250101, China; Joint SDU-NTU Centre for Artificial Intelligence Research (C-FAIR), Shandong University, Jinan 250101, China; School of Software, Shandong University, Jinan 250101, China; Joint SDU-NTU Centre for Artificial Intelligence Research (C-FAIR), Shandong University, Jinan 250101, China; School of Software, Shandong University, Jinan 250101, China; Joint SDU-NTU Centre for Artificial Intelligence Research (C-FAIR), Shandong University, Jinan 250101, China; Faculty of Applied Sciences, Macao Polytechnic University, Macao Special Administrative Region 999078, China

**Keywords:** ncPEP identification, Hypergraph learning, Peptide feature representation, Multi-label classification, Cancer biomarker

## Abstract

A small peptide encoded by a non-coding RNA (ncRNA), known as a non-coding peptide (ncPEP), is emerging as a critical regulator and biomarker in cancer, holding immense promise for immunotherapy. However, the systematic identification of ncPEPs remains a challenge because existing computational methods typically analyze peptides based on sequence alone. Sequence-only analysis overlooks the fundamental biological principle that multiple distinct peptides can be translated from a single non-coding RNA transcript, thus sharing a common transcriptional origin. Here, we address this limitation by developing HGCPep, a deep learning framework that leverages hypergraphs to model these intrinsic relationships. In our model, each ncRNA is represented as a hyperedge connecting the set of peptides it encodes, thereby enriching peptide feature representations with transcriptional context. We demonstrate that HGCPep, which integrates a hypergraph neural network with a convolutional neural network, outperforms state-of-the-art methods in identifying cancer-associated ncPEPs. Furthermore, dimensionality reduction of the learned embeddings reveals distinct clustering of ncPEPs by cancer type, illustrating how the model effectively deciphers complex biological associations. Our work introduces a new method for ncPEP analysis and provides a powerful tool for discovering novel therapeutic targets in oncology. The dataset and source code of our proposed method can be found via https://github.com/Longwt123/HGCPep_Github.

## Graphical abstract

**Figure:**
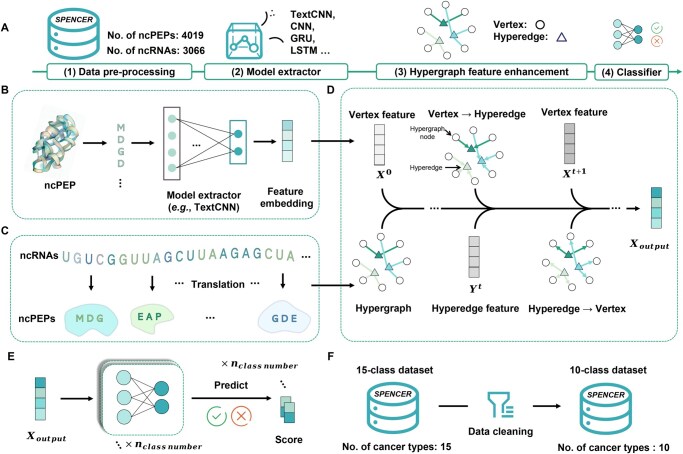

## Introduction

Traditionally, non-coding RNAs (ncRNAs) were presumed to lack coding potential, and thus earlier investigations focused primarily on their functional roles rather than their coding abilities [1]. However, recent breakthroughs in ncRNA research have revealed the presence of ncRNA-encoded small peptides (ncPEPs), which have opened new avenues in cancer research, particularly in the fields of immunotherapy and biomarker identification [2]. Recent breakthroughs in proteomics have identified short open reading frames (sORFs) within molecules previously deemed non-coding, leading to the discovery of peptides (ncPEPs) with diverse and potent functions [3–9]. This revelation has fundamentally redefined the functional landscape of ncRNAs, spurring investigations into their translational capacity alongside their established regulatory roles.

Within the realm of cancer research, the discovery of ncPEPs has introduced novel avenues for the identification of therapeutic targets and biomarkers, particularly in the context of cancer immunotherapy [10]. Cancer immunotherapy harnesses the inherent capabilities of the immune system to selectively target and eliminate cancer cells expressing specific antigens. Central to this approach are neoantigens, which arise from genetic alterations in tumor cells and can be recognized by T cell receptors (TCRs), triggering a highly specific anti-tumor immune response [11]. These ncPEPs hold promise as unique neoantigens that could be exploited to develop immunotherapeutic strategies with enhanced specificity and efficacy. Noteworthy examples of ncPEPs as potential biomarkers in cancer have also come to the fore. Chakraborty et al. reported consistent expression of ncPEPs across 11 carcinoma cell lines, suggesting their potential as generalizable biomarkers with remarkable stability [12]. Unraveling the expression patterns and functional implications of ncPEPs in different cancer types holds fundamental importance in deciphering the intricate pathogenesis of cancer and providing crucial insights for precision medicine and targeted therapies.

While protein mass spectrometry (MS) [13–15] remains the primary experimental technique for detecting ncPEPs, computational methods play a crucial role in complementing and enhancing these efforts. However, computational approaches face several challenges that must be overcome to fully exploit the potential of ncPEPs research in cancer. One significant challenge is the accurate association of ncPEPs with specific cancer types, necessitating the development of robust computational frameworks capable of deciphering the intricate relationships between ncPEPs and the molecular landscapes of diverse cancer subtypes. Furthermore, most peptide prediction functions solely rely on the sequence order, such as DeepPep [16] and AlphaPeptDeep [17]. However, they often overlook the inherent information, leading to ineffective and unreliable predictions. In line with the central dogma, which elucidates the translation of peptides from RNA through a many-to-many mapping, the majority of peptides in cancer research are found intracellularly. Leveraging this translation relationship within this context, we can enhance the robustness of our predictions. To harness this additional information, we propose the integration of hypergraphs into our model to better capture this relationship.

In recent years, the utilization of hypergraphs has demonstrated remarkable efficacy in modeling and capturing intricate correlations. The concept of hypergraph learning, initially introduced in [18], can be viewed as a transductive learning process that propagates through the hypergraph structure. This approach has witnessed significant advancements and widespread applications across various domains. In the context of tag-based image retrieval, Wang et al. [19] constructed a sophisticated hypergraph that incorporates global and local visual features alongside tag information to facilitate image relevance learning. This framework leverages the power of hypergraphs to capture complex relationships and enhance the retrieval performance. Building upon the unprecedented achievements of deep learning, researchers have extended hypergraph learning techniques to encompass deep architectures. For instance, Feng et al. [20] introduced hypergraph neural networks (HGNNs) to model and learn complex correlations that extend beyond pairwise relationships. In contrast to traditional graph neural networks (GNNs) [21], HGNNs employ a vertex-hyperedge-vertex information propagation scheme, enabling iterative learning of data representations within the hypergraph structure. The development of deep hypergraph learning techniques exemplifies the fusion of hypergraphs and deep learning, offering enhanced capabilities for capturing intricate correlations and optimizing data representations.

Here, we introduce HGCPep, a deep learning framework that leverages hypergraphs to model the biological relationship between ncRNA transcripts and the peptides they encode. In our framework, we represent peptides as nodes and model each ncRNA transcript as a hyperedge that connects the entire cohort of peptides it encodes. This design allows us to enrich the feature representation of each peptide with its crucial transcriptional context. By integrating a hypergraph neural network with a convolutional neural network, HGCPep learns to decipher complex biological associations that link ncPEPs to specific cancer types. Our results demonstrate that this approach not only outperforms existing methods but also generates biologically meaningful embeddings that reveal distinct clustering patterns by cancer type. Our work thus provides a powerful new tool for exploring the non-coding proteome and introduces a more biologically informed methodology for peptide function prediction.

## Method

### Datasets

**Figure 1:**
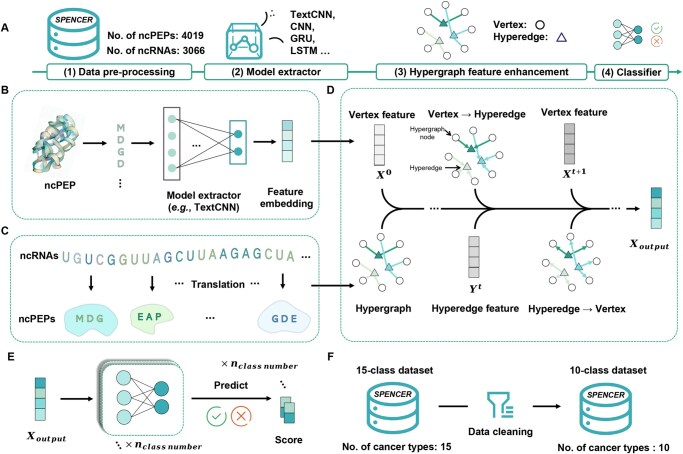
The framework of HGCPep method for ncPEP prediction **A**. The overall workflow schema of the HGCPep method for ncPEP prediction. It is composed of four blocks: (1) data pre-processing block, (2) model extractor block, (3) hypergraph feature enhancement block, and (4) classifier block. **B**. Hypergraph features extracted by the model extractor block. **C**. Hypergraphs constructed according to the correspondence between ncRNA and ncPEP. **D**. Feature representation strengthened by the hypergraph feature enhancement block. The circles represent hypergraph nodes, the triangles denote hyperedges, *X* represents the features of nodes, and *Y* represents the features of hyperedges. **E**. The classifier used to identify the association of a ncPEP with a specific type of cancer. **F**. The constructed datasets. ncPEPs, non-coding RNA-encoded peptides. ncRNAs, non-coding RNAs. ncPEP, non-coding RNA-encoded peptide; TextCNN, text convolutional neural network. CNN, convolutional neural network. GRU, gated recurrent unit. LSTM, long short-term memory.

The dataset of cancer-associated ncPEPs was constructed based on the SPENCER database [22], which contains 29,526 ncPEPs spanning 15 cancer types, of which 22,060 have been experimentally validated. We processed all entries from SPENCER, curating for each peptide its amino acid sequence and a corresponding list of associated cancer types. Given that a single peptide can be linked to multiple cancer types, we formulated the predictive task as a multi-label classification problem. A summary of the dataset statistics is provided in **Table 1** and **Figure 1**.

**Table 1:** Sample size statistics for the 15- and 10-class datasets

Cancer type	15-class dataset	10-class dataset
Anal canal cancer	518	518
Bile duct cancer	442	–
Bladder cancer	1178	1178
Breast cancer	1075	1075
Colon cancer	2068	2068
Gastric cancer	460	–
Kidney cancer	905	905
Leukemia	506	506
Liver cancer	244	–
Lung cancer	1646	1646
Ovary cancer	258	–
Prostate cancer	1028	1028
Skin cancer	552	552
Thyroid cancer	200	–
Tongue cancer	519	519

*Note*: There are a total of 4019 peptides and 3066 RNAs in both datasets. “–” indicates that the cancer type was excluded from the 10-class dataset.

### Hypergraph construction and data preprocessing

We constructed hypergraphs to model the biological relationships between ncRNAs and their translated products. In this framework, each peptide is represented as a node, and each ncRNA transcript is modeled as a hyperedge that connects the entire cohort of peptides it encodes. This design naturally captures the one-to-many relationship from transcript to peptides. Furthermore, it inherently accommodates the biological reality that a single peptide (node) can be translated from multiple ncRNAs, thus associating it with multiple hyperedges. This hypergraph structure provides a powerful representation of the complex, higher-order correlations within the ncPEP-ncRNA system.

The original peptide data includes the serial number, sequence information, and corresponding cancer label information of ncPEPs. First, it was mapped into psDict ={pid:sequence} and plDict = {pid: labels} dictionaries, where pid is the sequence number of ncPEPs, sequence is its sequence information, and labels is its label information. The original RNA data includes the sequence information of ncPEPs and the number of corresponding RNAs. We counted the set of RNA numbers by rDict={rid: frequency}, where rid is the sequence number of RNA and the frequency denotes how often each RNA appears in the original RNA data. Then, the data were mapped into cDict={pid:edges} using psDict, where edges=[rid1,rid2…] denote the list of RNAs corresponding to pid.

Next, we mapped each ncPEP to the vertices of the hypergraph while recording the number of vertices $num_{\mathrm{vertices}}$ and generating corresponding tags through plDict. We constructed each RNA hyperedge by rDict and cDict, concatenating information about corresponding vertices. Finally, a complete hypergraph was constructed by $G=\mathrm{Hypergraph}(num_{\mathrm{vertices}},\;\mathrm{edges})$, where Hypergraph is the function for constructing the hypergraph and G is the hypergraph we constructed (20), which was used for the downstream tasks.

### Dataset splitting

Both datasets were partitioned into training (80%), validation (10%), and independent test sets (10%). Models were trained on the training set, and the model with the highest performance on the validation set was selected as the optimal model. The generalization performance of this optimal model was then evaluated on the held-out test set.

### Framework overview

Within this section, we offer a brief overview of our framework, HGCPep. HGCPep is designed for representation learning and predicting the cancer type associated with ncPEPs using raw ncPEP sequences and corresponding RNA data. The flowchart of our framework is shown in Figure 1A, which includes four steps: (1) data pre-processing block, (2) model extractor block TextCNN (text convolution neural network) [23], (3) hypergraph feature enhancement block (20, 24), and (4) classifier block. For peptide sequence data, the TextCNN module is first used to facilitate feature extraction, extracting key features of the peptide sequence, as shown in Figure 1B. Subsequently, the innovative introduction of the hypergraph structure further enhances the exploration of the potential relationship between peptides and RNA, where the construction of the hypergraph is shown in Figure 1C and the application of hypergraph convolution is shown in Figure 1D. The hypergraph convolution [24] is then applied to elevate the correlation features to generate a more robust representation of our problem. Finally, the latent features are obtained through the fusion of key features and association features of peptide sequences and are then utilized by a multi-label multi-class classifier to predict the corresponding cancer types (Figure 1E). In addition, Figure 1F shows the two data sets we used, which contain data from 15 and 10 classes, respectively.

### HGNNP module

A fundamental challenge in ncPEP analysis lies in modeling the complex biological relationships that extend beyond simple pairwise interactions. While conventional graphs, with edges connecting only two nodes, are inadequate for this task, hypergraphs provide a natural and powerful mathematical framework. A hypergraph permits an edge — termed a hyperedge — to connect an arbitrary number of nodes, making it ideally suited for representing the group relationships inherent in our data: the “one-to-many” mapping from a single ncRNA transcript to the multiple peptides it encodes.

Therefore, we adopted a hypergraph-based approach to explicitly model this biological context. First, we collated the dataset and recorded the number of peptides as the number of hypergraph vertices. We then extracted the available paired data correlations and constructed the hyperedge data accordingly. Here we let $G_{s}=(V_{s},E_{s})\;$indicate the hypergraph structure, where $v_{i}\in V_{s}$ is a vertex and $e_{s_{ij\cdots n}}\in E_{s}$ is a hyperedge in the hypergraph connecting $v_{i},v_{j},\ldots ,v_{n}$, where $e_{s_{ij\cdots n}}$ is denoted by $e_{s_{ij\cdots n}}=(v_{i},v_{j},\ldots ,v_{n})$ and $E_{s}$ is expressed as:


(1) Es={(vi,vj,…,vn),(vk,vl,…,vm),…}

Let N denote the number of hypergraph vertices. Given such N and $E_{s}$, the hypergraph $G_{s}\;$can be generated as follows:


(2) Gs=Hypergraph(N,Es)

Next, Gao et al. proposed the hypergraph convolution model HGNNConv+ [24]. According to the DHG toolkit documentation, we first establish a general hypergraph convolution layer known as HGNNP. This innovative layer comprises a two-phase messaging framework that contains two-phase aggregation functions. In HGNNP, there are two model lists named HGNNPConv, each containing fully connected layers, normalization layers, feature update layers, and dropout layers. The feature update layers include an aggregation-update layer from nodes to edges and an aggregation-update layer from edges to nodes, similar to GNNs. These structures enable HGNNP to be more flexible and perform well in downstream tasks.

The details of HGNNP utilized in our model can be observed in the formula (3), where $X^{t}$ represents the feature set of input vertices at layer t. The transpose of H empowers the control of each vertex feature in the $X^{t}$ with respect to its hyperedge neighbors. Therefore, it can be used to guide the aggregation of each vertex, ultimately producing the hyperedge feature set $Y^{t}$, which can be represented mathematically as $Y^{t}=WD_{e}^{-1}H^{⊤}X^{t}$. The updating of the vertex feature set $X^{t+1}$ from the hyperedge feature set $Y^{t}$ can be expressed as $X^{t+1}=\sigma (D_{v}^{-1}HY^{t}\Theta^{t})$. Therefore, the matrix representation of HGNNP can be denoted as:


(3) Xt+1=σ(Dv-1HWDe-1H⊤XtΘt)

### Text convolution neural network

The text convolution neural network (TextCNN) [23] shares similarities with the convolution neural network (CNN) [25] structure. It is a modified version of CNN that leverages the parallel computing capabilities of CNN to accelerate the training process. In addition to preserving the original characteristics of CNN, TextCNN also incorporates text feature extraction, making it applicable for extracting peptide sequence features in our study. The resemblance between text and peptide sequence allows TextCNN to effectively identify linguistic n-grams in the task. Moreover, its convolution structure enables the sharing of predicted behavior among n-grams that have similar elements, even if a particular n-gram has not been encountered during prediction. Consequently, the multi-channel convolution mechanism in TextCNN automatically captures both local and global information from the sequence, facilitating the learning of peptide sequence features specific to our task.

### Performance measurement

Following previous works, we evaluated our model using accuracy (ACC), Matthews correlation coefficient (MCC), precision (PREC), recall (REC), and area under the ROC curve (AUC). ACC is the ratio of correctly classified samples to the total, providing an intuitive performance measure. PREC indicates the proportion of positive predictions that are correct, while REC measures the fraction of actual positives correctly identified. MCC balances performance across all classes in the confusion matrix, making it reliable for imbalanced datasets. AUC, ranging from 0 to 1, represents the probability that a randomly selected positive sample is ranked higher than a negative one by the model, with 0.5 equivalent to random guessing.

## Results

### Hypergraph integration improves ncPEP prediction

**Figure 2:**
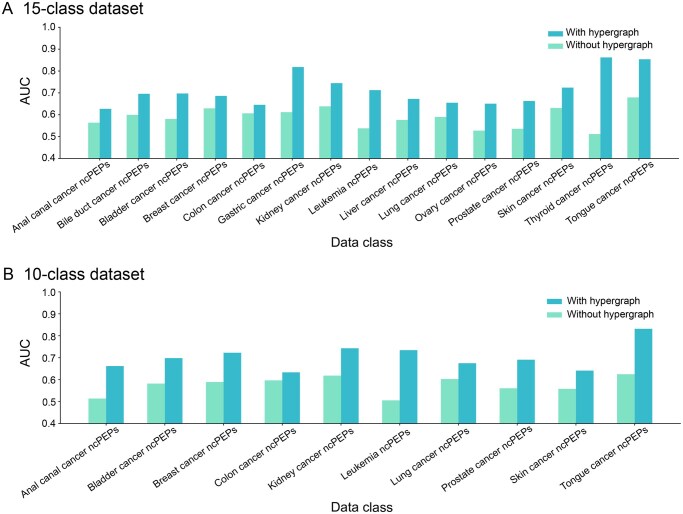
Performance evaluation on the accuracy of HGCPep Performance evaluation on the accuracy of HGCPep for predicting ncPEPs in various types of cancer in the 15-class dataset (**A**) and the 10-class dataset (**B**). AUC, area under the receiver operating characteristic curve.

To test the effectiveness of HGCPep on the prediction of multi-label classification problems, we conducted a comprehensive analysis of model prediction AUC scores for each ncPEP class corresponding to different cancer types, as shown in **Figure 2**. We used HGCPep as the base model. The prediction AUC of all classes was improved overall after the hypergraph information was incorporated into the model on both datasets. Being 37.92% higher than the model without hypergraphs, the thyroid cancer class exhibited the most noticeable improvement in the 15-class dataset. Notably, for the leukemia class within the 10-class dataset, our hypergraph-based model achieved a 22.88% improvement in AUC over the baseline model. These results suggest that the hypergraph structure and the hypergraph convolution module play a critical role in improving the prediction accuracy of each class.

**Figure 3:**
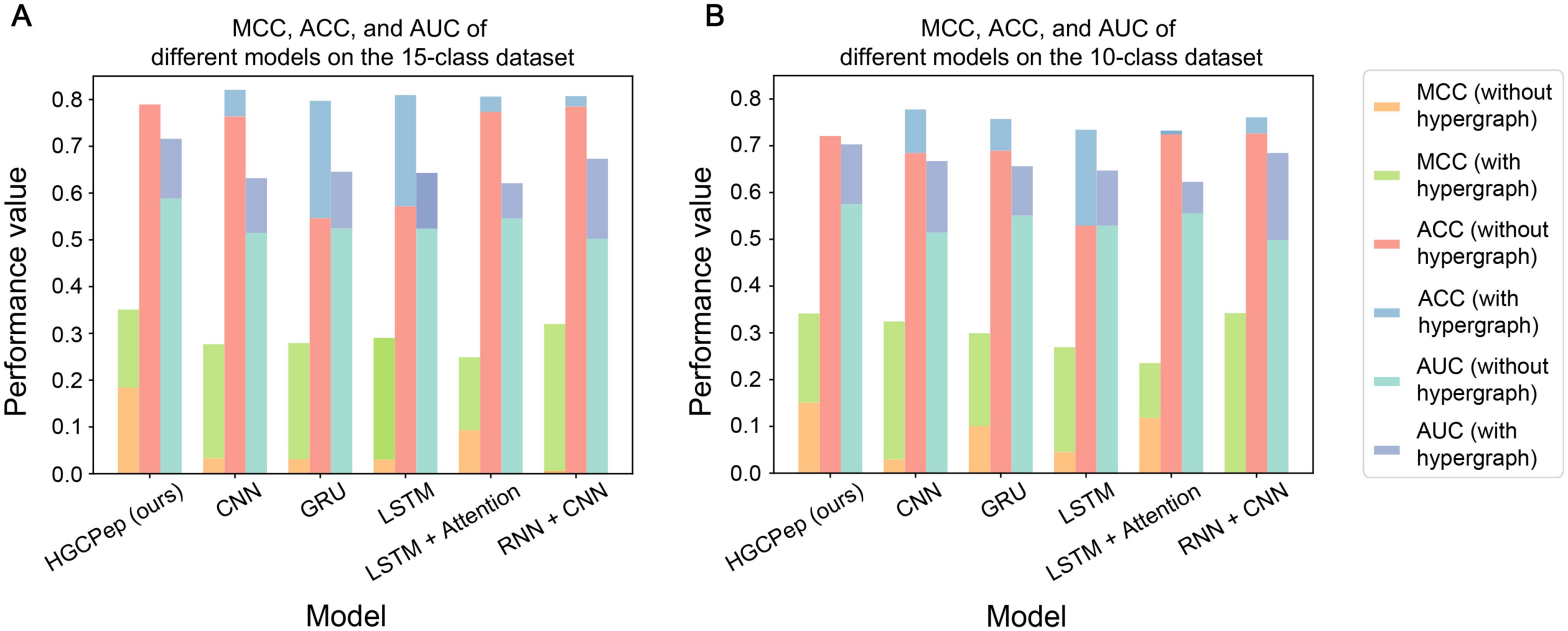
Performance evaluation on the MCC, ACC, and AUC of HGCPep Performance evaluation on the MCC, ACC, and AUC of HGCPep, CNN, GRU, LSTM, LSTM with Attention, RNN, and CNN for predicting ncPEPs in the 15-class dataset (**A**) and the 10-class dataset (**B**). ACC, accuracy. MCC, Matthews correlation coefficient. RNN, recurrent neural network.

To test whether the enhanced capability of hypergraph is limited only to our model, we combined hypergraph convolution with other commonly used embedding models and compared their prediction performance. Here, we chose some popular methods, including CNN [25], gated recurrent unit (GRU) [26], long short-term memory (LSTM) [27], LSTM with attention [28], as well as a hybrid model combining a CNN with a recurrent neural network (RNN) [29], hereafter referred to as CNN+RNN. Through the comparison of MCC, ACC, and AUC metrics in **Figure 3**, we observed that integrating hypergraph-derived information enhanced the feature embeddings of the baseline models. This integration consistently led to substantial performance improvements across all tested architectures. The integration of the hypergraph module consistently enhanced the performance across all baseline models (Figure 3). On the 15-class dataset, this led to mean improvements of 9.72%, 23.16%, and 12.22% in MCC, ACC, and AUC, respectively (Figure 3A). Similarly, on the 10-class dataset, we observed average gains of 6.65%, 20.91%, and 12.64% for the same metrics (Figure 3B). Models without hypergraphs exhibited high ACC scores, but their precision scores were notably low. These findings demonstrate that hypergraphs excel at modeling the latent, high-dimensional correlations between ncPEPs and their corresponding ncRNAs. This, in turn, leads to a richer and more informative feature representation.

### Performance comparison of different hypergraph convolution models

**Figure 4:**
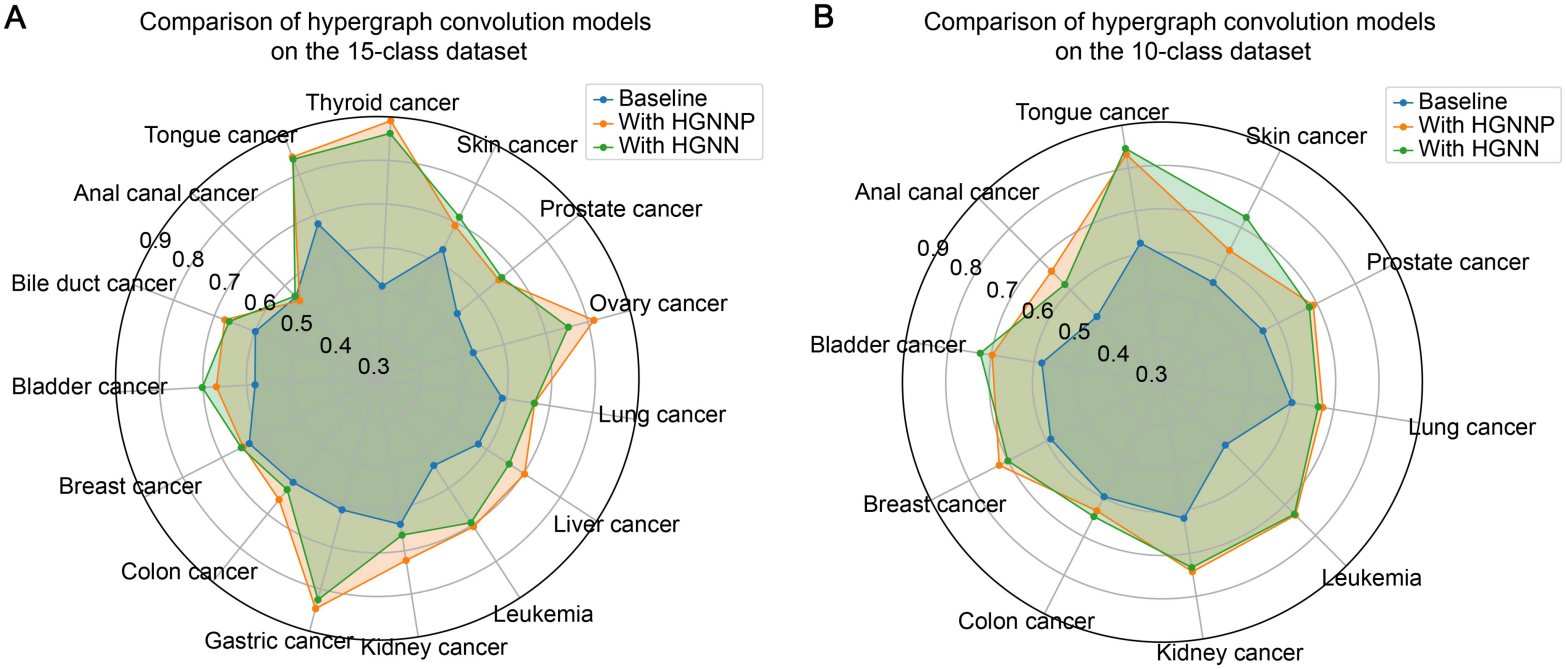
Performance evaluation on the AUC of the various hypergraph modules Performance evaluation on the AUC of the various hypergraph modules employed by HGCPep for predicting ncPEPs associated with cancers in the 15-class dataset (**A**) and the 10-class dataset (**B**). HGNN, hypergraph neural networks. HGNNP, hypergraph neural networks plus.

To compare the influence of different types of hypergraph convolution on prediction results, we employed TextCNN as the basic model and conducted comparative experiments with the HGNN hypergraph convolution model and the HGNNP hypergraph convolution model according to the study of Gao and colleagues [24]. The comparison results of AUC are shown in **Figure 4** below. From these results, we could make the following observations: (1) Hypergraphs improved the model prediction performance. The average performance improvements of the two hypergraph convolution models were 12.80% and 11.56% in the 15-class dataset, with a peak of 37.92% for thyroid cancer ncPEPs. Improvements were also made in the 10-class dataset. (2) In comparison to HGNN and HGNNP hypergraph convolutions, HGNNP demonstrated improved performance across 6 classes, similar performance in 7 classes (with differences not exceeding ± 2.0%), and decreased performance in 2 classes within the 15-class dataset. For the 10-class dataset, the corresponding numbers are 2, 6, and 2 classes, respectively. In conclusion, the HGNNP hypergraph convolution module is more effective for the prediction of ncPEPs of cancer compared to the HGNN module.

### Performance comparison of different graph convolution models

**Figure 5:**
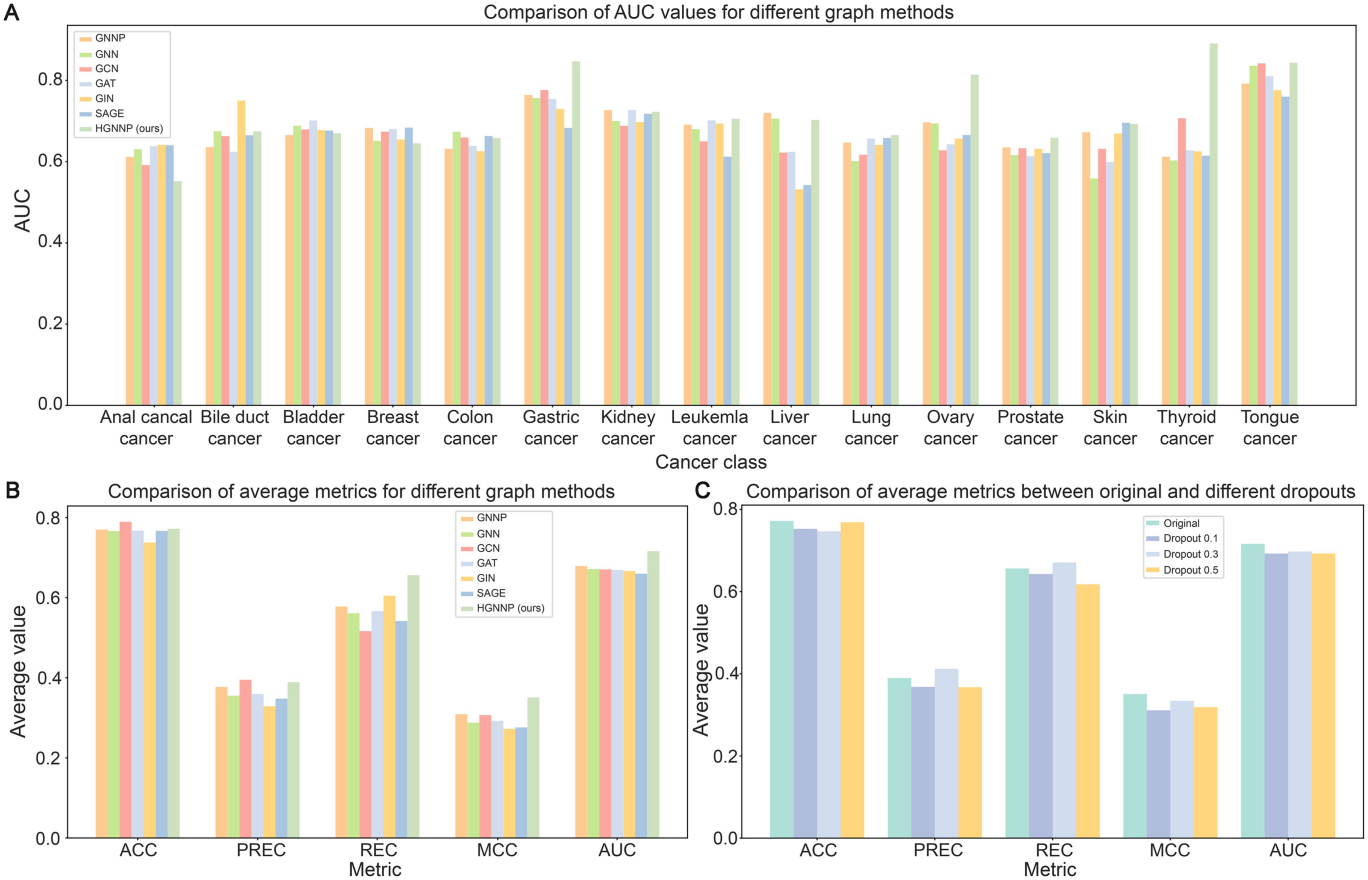
Performance evaluation of the various graph modules for predicting ncPEPs **A**. Comparison of AUC values for different graph methods across 15 cancer-associated ncPEPs. **B**. Comparison of average ACC, PREC, REC, MCC, and AUC values for different graph methods. **C**. Performance evaluation of different dropout parameters. GNNP, graph neural network plus. GNN, graph neural network. GCN, graph convolutional network. GAT, graph attention network. GIN, graph isomorphism network. SAGE, sample, and aggregation. HGNNP, hypergraph neural networks plus. PREC, precision. REC, recall.

To compare the impact of different types of graph convolutions on the prediction results, we used TextCNN as the base model and conducted comparative experiments with classic graph neural network models based on the study by Huang and colleagues [30]. As **Figure 5**A and B illustrate, our hypergraph model outperformed the classic ordinary graph models. Additionally, we investigated potential overfitting of the model by comparing models with different dropout parameters, confirming that our model did not exhibit significant overfitting (Figure 5C).

### Performance comparison between different loss functions

**Figure 6:**
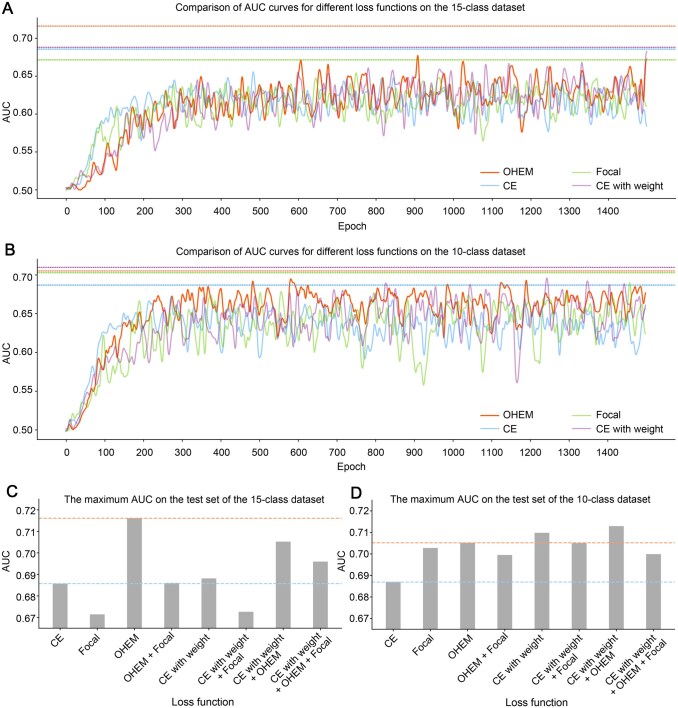
Performance evaluation on the AUC of HGCPep **A**. Comparison of AUC curves for different loss functions on the 15-class dataset. **B**. Comparison of AUC curves for different loss functions on the 10-class dataset. **C**. Comparison of the maximum AUC values for different loss functions on the 15-class dataset. **D**. Comparison of the maximum AUC values for different loss functions on the 10-class dataset. OHEM, online hard example mining. CE, cross-entropy loss.

To overcome the imbalance in multi-label classification of ncPEPs, we employed several special loss functions in the HGCPep model’s training process. Here, we compared the performance of different loss functions for optimizing HGCPep, including Online Hard Example Mining (OHEM) [31], commonly used Focal Loss, Cross Entropy Loss (CE), and CE with weight. By recording and comparing the AUC indexes of different losses in the training process, we compared the performance of HGCPep under different loss functions, as shown in **Figure 6**. It should be noted that the curves were smoothed using Gaussian smoothing. As shown in Figure 6A and B, the training process of our model on the two datasets was stable without large fluctuations, and the overall effect was good. The bar plot in Figure 6C compares the maximum AUC values obtained with different loss functions, illustrating the exceptional performance of the OHEM loss in the 15-class dataset. With the dataset cleaned to reduce its imbalance, the prominence of the OHEM loss diminished in the 10-class dataset, as depicted in Figure 6B. To better learn which loss function works in the two datasets, we compared different combinations of three loss functions (Figure 6C and D). The impact of the OHEM technique on the imbalanced dataset was more pronounced in the 15-class dataset compared to the 10-class dataset. In the imbalanced dataset, various loss functions exhibited unstable performance, making it challenging to determine the optimal choice. However, in the 10-class dataset, all specialized loss functions demonstrated improvement over the original CE loss, indicating the effectiveness of this dataset for deep learning training applications.

### Visualization of HGCPep

**Figure 7:**
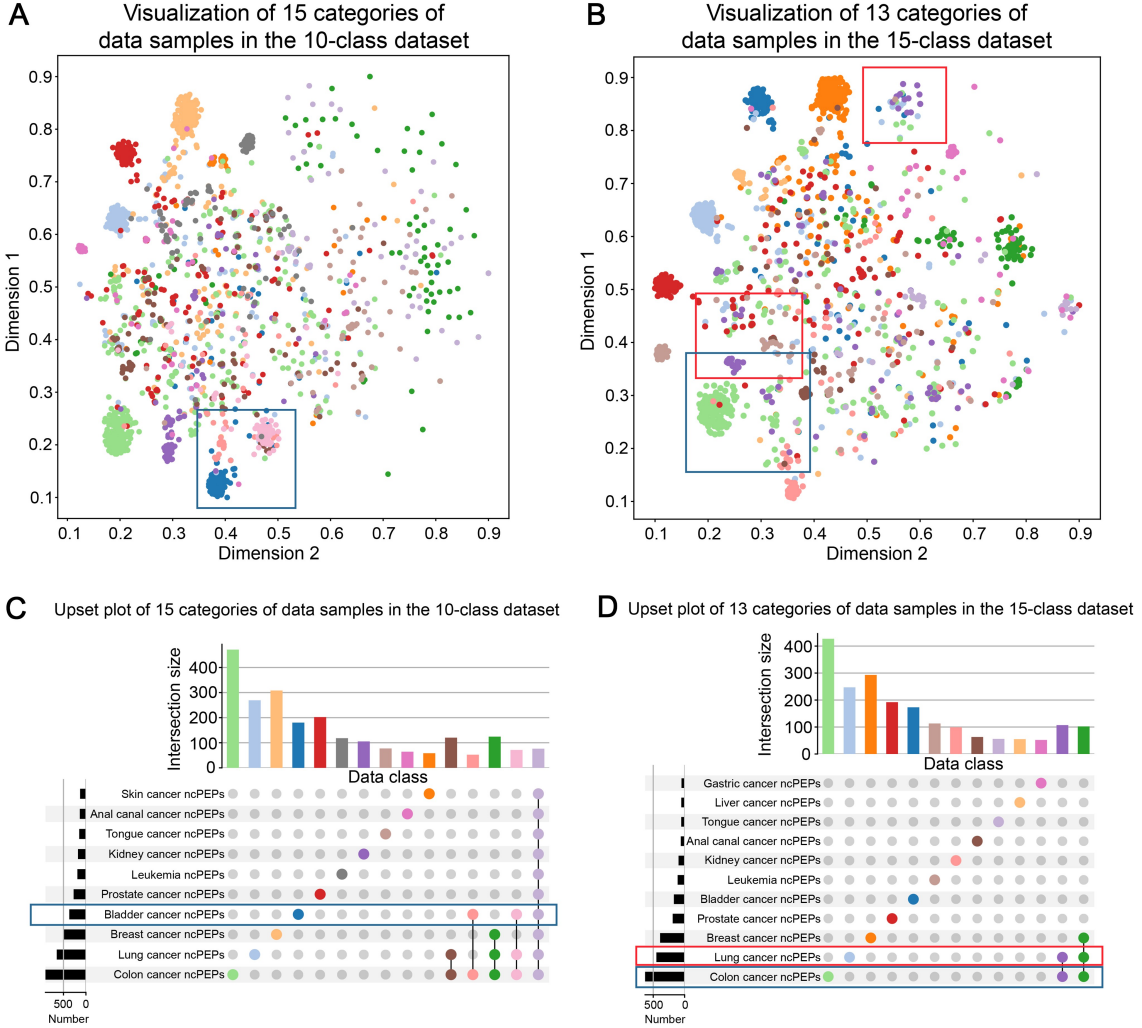
Evaluating HGCPep in Euclidean space **A**. Visualization of data samples in Euclidean space for the 10-class dataset. **B**. Visualization of data samples in Euclidean space for the 15-class dataset. **C**. Upset plot of multi-label category distributions in the 10-class dataset. **D**. Upset plot of multi-label category distributions in the 15-class dataset. In (A) and (C), the differently colored dots and bars represent cancer categories, with colors corresponding consistently within (B) and (D). In the tables shown in (C) and (D), each row represents a cancer label, with the bar on the left indicating the number of instances; each column represents a multi-label combination, with the bar on top indicating the number of instances.

Both datasets we used inherently exhibited a hypergraph structure, reflecting the complex higher-order correlations among ncPEP samples. To illustrate the learning capability of the hypergraph-based methods, we projected the output of the optimal model of HGCPep into Euclidean space [24], and the results are shown in **Figure 7**. In Figure 7A and B, different colors represent different multi-label categories of data, and their dimensions were reduced to two. The multi-label categories represented in Figure 7C correspond to those in Figure 7A. Each row of Figure 7D corresponds to one class in the 15-class dataset, each column to a multi-label category containing one or more points representing multiple labels, and the bar graphs indicate the number of samples in each category. The color scheme is consistent across related panels. The results showed that our hypergraph-based method produced discernible clustering, qualitatively verifying its effectiveness.

For the visualization results in the 10-class dataset, in the highlighted region within the blue box of Figure 7A, data points labeled in blue, light pink, and pink tended to cluster together, suggesting that they share a common label of bladder cancer, which was further supported by the bar graphs (the seventh row in Figure 7C). As for the dark green and light purple labeled data points scattered in the figure, we speculate that their difficulty in achieving visible clustering stems from their involvement with three different label categories and similarity to many other labeled data points. Consequently, the model faces challenges in accurately classifying them, leading to less distinct visual clustering effects.

Regarding the visualization results in the 15-class dataset, the partial region highlighted in the red box in Figure 7B demonstrated the clustering results of the purple-labeled data and light blue-labeled data, which exhibited a close resemblance. Through careful analysis and statistical examination, these data points shared a common label of lung cancer, resulting in similar clustering outcomes after dimensionality reduction, as indicated in the ninth row of Figure 7D. Similarly, in the blue box, the purple-labeled data and light green-labeled data exhibited proximity in their clustering results and shared the common label of colon cancer. In addition, we conducted other visualization methods [32] and comparative experiments [33–35], as detailed in Figure S2–S5.

## Discussion

In this study, we introduced HGCPep, a deep learning framework that utilizes hypergraphs to model the intrinsic biological relationship between ncRNAs and multiple peptides they encode. Our approach successfully addresses the fundamental limitation of existing computational methods, which typically analyze peptides in isolation based on sequence alone. HGCPep outperforms other models in identifying cancer-associated ncPEPs, thereby establishing a new and more biologically informed paradigm for peptide function prediction.

The primary innovation of our work lies in the explicit integration of biological context — the shared transcriptional origin — into the feature learning process. The success of HGCPep is rooted in its ability to capture these higher-order, many-to-many relationships, which are intractable for conventional graph models limited to pairwise connections. Our hypergraph representation, where each ncRNA transcript acts as a hyperedge connecting its cohort of encoded peptides (nodes), enriches the feature embeddings of individual peptides with crucial contextual information. This enriched representation allows the model to learn not just from “what” a peptide’s sequence is, but also “where” it comes from. The finding that the hypergraph module serves as a versatile enhancement component, consistently boosting the performance of various baseline architectures, underscores the universal value of this contextual information and the robustness of our approach.

In addition to superior predictive accuracy, the learned embeddings from HGCPep offer valuable biological insights. The distinct clustering of ncPEPs by cancer type in a reduced-dimensional space suggests that our model is deciphering meaningful biological signatures that link peptide origins to specific cancer pathologies. This capability transforms HGCPep from a mere classification tool into a model for analysis, enabling researchers to explore potential functional roles and biomarker panels for ncPEPs based on their learned proximity in the embedding space.

Our work also extends beyond standard GNNs, which, despite their success in modeling biological networks, are fundamentally constrained to pairwise interactions and cannot naturally represent group relationships. The empirical superiority of the HGNNP module over the standard HGNN in our experiments further highlights that the specific choice of hypergraph convolution architecture is critical for effectively propagating information in this unique problem setting. Moreover, our systematic evaluation of different loss functions, which confirms the utility of OHEM for handling imbalanced multi-label data, provides a practical guide for future studies in this domain.

Despite its strengths, our study has several limitations that open avenues for future research. First, our model is currently trained on data from the SPENCER database. While comprehensive, any single database may have inherent biases in data collection and annotation. Future work should aim to integrate data from more diverse sources, including large-scale proteomics, ribosome profiling (Ribo-seq), and other experimental assays to build more comprehensive and robust models. Second, our hypergraph models a single type of biological relationship: a shared transcriptional origin. Biological systems, however, are governed by a multitude of intersecting relationships. Future iterations could involve creating multi-relational or multi-modal hypergraphs that incorporate other layers of information, such as shared protein domains, subcellular localization, or protein-protein interactions, to generate even richer representations. Finally, as a computational study, our predictions require experimental validation. The candidate cancer-associated ncPEPs identified by HGCPep should be validated by wet-lab experiments to confirm their expression, function, and potential as therapeutic targets or diagnostic biomarkers.

In conclusion, our work demonstrates that hypergraphs provide a powerful and natural framework for modeling the complex biology of ncPEPs. By moving beyond sequence-only analysis, HGCPep paves the way for a new generation of computational tools that can more accurately decipher the roles of the non-coding proteome in health and disease.

## Conclusion

In this study, we present HGCPep, an innovative deep-learning framework that successfully integrates fundamental biological principles with advanced network science to identify cancer-associated ncPEPs. By representing the shared transcriptional origin of peptides as hyperedges in a graph, our model captures complex, higher-order relationships that are overlooked by existing methods. HGCPep not only achieves state-of-the-art performance in multi-label classification across diverse cancer types but also generates biologically meaningful embeddings. This work provides a powerful and robust tool for discovering novel biomarkers and therapeutic targets in oncology and establishes a paradigm for modeling complex relationships in genomics and proteomics, paving the way for more sophisticated multi-omics integration in the future.

## Code availability

The source code for the HGCPep model and the datasets used in our work is publicly available at the GitHub repository (https://github.com/Longwt123/HGCPep_Github). It has also been submitted to BioCode at the National Genomics Data Center (NGDC), China National Center for Bioinformation (CNCB) (BioCode: BT007777), which is publicly accessible at https://ngdc.cncb.ac.cn/biocode/tool/BT7777.

## CRediT author statement

**Wentao Long:** Conceptualization, Methodology, Software, Validation, Formal analysis, Investigation, Writing – original draft, Writing – review & editing, Visualization. **Zhongshen Li:** Conceptualization, Methodology, Investigation, Resources, Data curation, Writing – original draft, Writing – review & editing, Supervision, Project administration. **Junru Jin:** Methodology, Formal analysis, Writing – original draft, Writing – review & editing, Supervision, Project administration. **Jianbo Qiao:** Writing – review & editing, Supervision, Project administration. **Yu Wang:** Supervision. **Leyi Wei:** Supervision, Project administration, Funding acquisition. All authors have read and approved the final manuscript.

## Competing interests

The authors have declared no competing interests.

## Supplementary Material

qzaf093_Supplementary_Data
