# Supplementary material for: HGCPep: Hypergraph Deep Learning Identifies Cancer-associated Non-coding Peptides
Source: Genomics Proteomics Bioinformatics. 2025 Dec 2;23(6):qzaf093. doi: 10.1093/gpbjnl/qzaf093 (PMC13183667; doi:10.1093/gpbjnl/qzaf093)
Supplement: qzaf093_Supplementary_Data [file qzaf093_supplementary_data.zip › Table S8.docx]

**Table S8 Precision and Recall of all models on the 10-class dataset**

|  | **Without HyperGraph** | | **With HyperGraph** | |
| --- | --- | --- | --- | --- |
|  | **PREC** | **REC** | **PREC** | **REC** |
| CNN | 0.2567 | 0.2472 | 0.4705 | 0.5057 |
| GRU | 0.3116 | 0.3279 | 0.4450 | 0.5137 |
| LSTM | 0.2523 | 0.5746 | 0.4027 | 0.5391 |
| LSTM with Attention | 0.3353 | 0.2965 | 0.4107 | 0.4304 |
| RNN and CNN | 0.2119 | 0.1487 | 0.4631 | 0.5788 |
| **HGCPep (ours)** | 0.3452 | 0.3613 | 0.4071 | 0.7213 |
