# Supplementary material for: HGCPep: Hypergraph Deep Learning Identifies Cancer-associated Non-coding Peptides
Source: Genomics Proteomics Bioinformatics. 2025 Dec 2;23(6):qzaf093. doi: 10.1093/gpbjnl/qzaf093 (PMC13183667; doi:10.1093/gpbjnl/qzaf093)
Supplement: qzaf093_Supplementary_Data [file qzaf093_supplementary_data.zip › Supplementary material captions.docx]

**Supplementary material**

**Figure S1 Example of hypergraph construction on the both datasets**

**Figure S2 ROC for each class of HGCPep in the 15-class dataset**

**Figure S3 Precision–recall curve for each class of HGCPep in the 15-class dataset**

**Figure S4 ROC for each class of HGCPep in the 10-class dataset**

**Figure S5 Precision-Recall curve for each class of HGCPep in the 10-class dataset**

**Table S1 Performance evaluation on the accuracy of HGCPep for predicting ncPEPs in various types of cancers in the 15-class dataset**

**Table S2 Performance evaluation on the accuracy of HGCPep for predicting ncPEPs in various types of cancers in the 10-class dataset**

**Table S3 Performance metrics (MCC, ACC, AUC) on the 15-class dataset**

**Table S4 Performance metrics (MCC, ACC, AUC) on the 10-class dataset**

**Table S5 Performance evaluation of various hypergraph modules employed by HGCPep for predicting ncPEPs in various cancers in the 15-class dataset**

**Table S6 Performance evaluation of the various hypergraph modules employed by HGCPep for predicting ncPEPs in various cancers in the 10-class dataset**

**Table S7 Precision and Recall of all models on the 15-class dataset**

**Table S8 Precision and Recall of all models on the 10-class dataset**
