# Supplementary material for: HGCPep: Hypergraph Deep Learning Identifies Cancer-associated Non-coding Peptides
Source: Genomics Proteomics Bioinformatics. 2025 Dec 2;23(6):qzaf093. doi: 10.1093/gpbjnl/qzaf093 (PMC13183667; doi:10.1093/gpbjnl/qzaf093)
Supplement: qzaf093_Supplementary_Data [file qzaf093_supplementary_data.zip › Table S2.docx]

**Table S2 Performance evaluation on the accuracy of HGCPep for predicting ncPEPs in various types of cancers in the 10-class dataset.**

|  | **Without HyperGraph** | **With HyperGraph** |
| --- | --- | --- |
| Anal canal cancer | 0.5137 | 0.6619 |
| Bladder cancer | 0.5819 | 0.6980 |
| Breast cancer | 0.5891 | 0.7226 |
| Colon cancer | 0.5966 | 0.6333 |
| Kidney cancer | 0.6181 | 0.7430 |
| Leukemia | 0.5056 | 0.7344 |
| Lung cancer | 0.6028 | 0.6748 |
| Prostate cancer | 0.5606 | 0.6907 |
| Skin cancer | 0.5579 | 0.6411 |
| Tongue cancer | 0.6247 | 0.8317 |
