# Supplementary material for: HGCPep: Hypergraph Deep Learning Identifies Cancer-associated Non-coding Peptides
Source: Genomics Proteomics Bioinformatics. 2025 Dec 2;23(6):qzaf093. doi: 10.1093/gpbjnl/qzaf093 (PMC13183667; doi:10.1093/gpbjnl/qzaf093)
Supplement: qzaf093_Supplementary_Data [file qzaf093_supplementary_data.zip › Table S3.docx]

**Table S3 Performance metrics (MCC, ACC, AUC) on the 15-class dataset**

|  | **Without HyperGraph** | | | **With HyperGraph** | | |
| --- | --- | --- | --- | --- | --- | --- |
|  | **MCC** | **ACC** | **AUC** | **MCC** | **ACC** | **AUC** |
| CNN | 0.0331 | 0.7640 | 0.5149 | 0.2770 | 0.8209 | 0.0331 |
| GRU | 0.0308 | 0.5464 | 0.5240 | 0.2795 | 0.7972 | 0.0308 |
| LSTM | 0.0304 | 0.5716 | 0.5239 | 0.2905 | 0.8096 | 0.0304 |
| LSTM with Attention | 0.0933 | 0.7735 | 0.5449 | 0.2491 | 0.8063 | 0.0933 |
| RNN and CNN | 0.0059 | 0.7849 | 0.5025 | 0.3203 | 0.8071 | 0.0059 |
| **HGCPep (ours)** | 0.1841 | 0.7894 | 0.5881 | 0.3508 | 0.7720 | 0.7161 |
