# Supplementary material for: HGCPep: Hypergraph Deep Learning Identifies Cancer-associated Non-coding Peptides
Source: Genomics Proteomics Bioinformatics. 2025 Dec 2;23(6):qzaf093. doi: 10.1093/gpbjnl/qzaf093 (PMC13183667; doi:10.1093/gpbjnl/qzaf093)
Supplement: qzaf093_Supplementary_Data [file qzaf093_supplementary_data.zip › Table S4.docx]

**Table S4 Performance metrics (MCC, ACC, AUC) on the 10-class dataset**

|  | **Without HyperGraph** | | | **With HyperGraph** | | |
| --- | --- | --- | --- | --- | --- | --- |
|  | **MCC** | **ACC** | **AUC** | **MCC** | **ACC** | **AUC** |
| CNN | 0.0295 | 0.6846 | 0.5147 | 0.3244 | 0.7779 | 0.6675 |
| GRU | 0.1005 | 0.6896 | 0.5504 | 0.2991 | 0.7575 | 0.6564 |
| LSTM | 0.0452 | 0.5291 | 0.5292 | 0.2695 | 0.7343 | 0.6471 |
| LSTM with Attention | 0.1183 | 0.7244 | 0.5553 | 0.2356 | 0.7323 | 0.6230 |
| RNN and CNN | −0.0062 | 0.7264 | 0.4988 | 0.3423 | 0.7609 | 0.6846 |
| **HGCPep (ours)** | 0.1506 | 0.7209 | 0.5751 | 0.3416 | 0.7107 | 0.7031 |
