# Supplementary material for: HGCPep: Hypergraph Deep Learning Identifies Cancer-associated Non-coding Peptides
Source: Genomics Proteomics Bioinformatics. 2025 Dec 2;23(6):qzaf093. doi: 10.1093/gpbjnl/qzaf093 (PMC13183667; doi:10.1093/gpbjnl/qzaf093)
Supplement: qzaf093_Supplementary_Data [file qzaf093_supplementary_data.zip › Table S5.docx]

**Table S5 Performance evaluation of various hypergraph modules employed by HGCPep for predicting ncPEPs in various cancers in the 15-class dataset**

|  | **Baseline**  **(without HyperGraph)** | **With HGNN** | **With HGNNP** |
| --- | --- | --- | --- |
| Anal canal cancer | 0.5639 | 0.5666 | 0.5517 |
| Bile duct cancer | 0.5995 | 0.6639 | 0.6744 |
| Bladder cancer | 0.5806 | 0.7018 | 0.6695 |
| Breast cancer | 0.6293 | 0.6499 | 0.6452 |
| Colon cancer | 0.6066 | 0.6283 | 0.6579 |
| Gastric cancer | 0.6119 | 0.8254 | 0.8466 |
| Kidney cancer | 0.6387 | 0.6638 | 0.7225 |
| Leukemia | 0.5382 | 0.6945 | 0.7054 |
| Liver cancer | 0.5764 | 0.6606 | 0.7027 |
| Lung cancer | 0.5902 | 0.6644 | 0.6653 |
| Ovary cancer | 0.5277 | 0.7536 | 0.8142 |
| Prostate cancer | 0.5360 | 0.6671 | 0.6586 |
| Skin cancer | 0.6311 | 0.7146 | 0.6927 |
| Thyroid cancer | 0.5119 | 0.8622 | 0.8911 |
| Tongue cancer | 0.6793 | 0.8379 | 0.8434 |
