# Supplementary material for: HGCPep: Hypergraph Deep Learning Identifies Cancer-associated Non-coding Peptides
Source: Genomics Proteomics Bioinformatics. 2025 Dec 2;23(6):qzaf093. doi: 10.1093/gpbjnl/qzaf093 (PMC13183667; doi:10.1093/gpbjnl/qzaf093)
Supplement: qzaf093_Supplementary_Data [file qzaf093_supplementary_data.zip › Table S7.docx]

**Table S7 Precision and Recall of all models on the 15-class dataset**

|  | **Without HyperGraph** | | **With HyperGraph** | |
| --- | --- | --- | --- | --- |
|  | **PREC** | **REC** | **PREC** | **REC** |
| CNN | 0.2138 | 0.1734 | 0.4654 | 0.3820 |
| GRU | 0.1968 | 0.5420 | 0.4019 | 0.4620 |
| LSTM | 0.1944 | 0.4911 | 0.4342 | 0.4321 |
| LSTM with Attention | 0.2625 | 0.2191 | 0.4174 | 0.3709 |
| RNN and CNN | 0.1716 | 0.1039 | 0.4218 | 0.4944 |
| **HGCPep (ours)** | 0.3419 | 0.3235 | 0.3893 | 0.6561 |
