# Supplementary figures and images for: HGCPep: Hypergraph Deep Learning Identifies Cancer-associated Non-coding Peptides
Source: Genomics Proteomics Bioinformatics. 2025 Dec 2;23(6):qzaf093. doi: 10.1093/gpbjnl/qzaf093 (PMC13183667; doi:10.1093/gpbjnl/qzaf093)

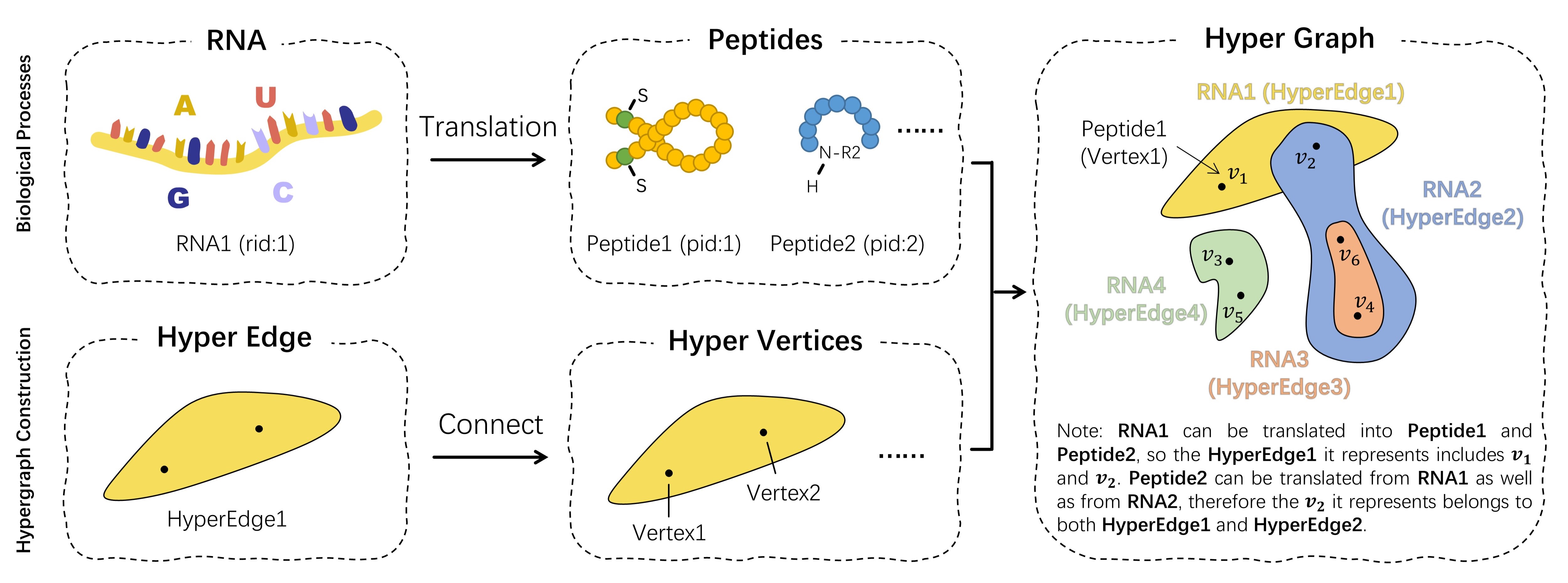

Supplement: qzaf093_Supplementary_Data [file qzaf093_supplementary_data.zip › Figure S1.jpg]

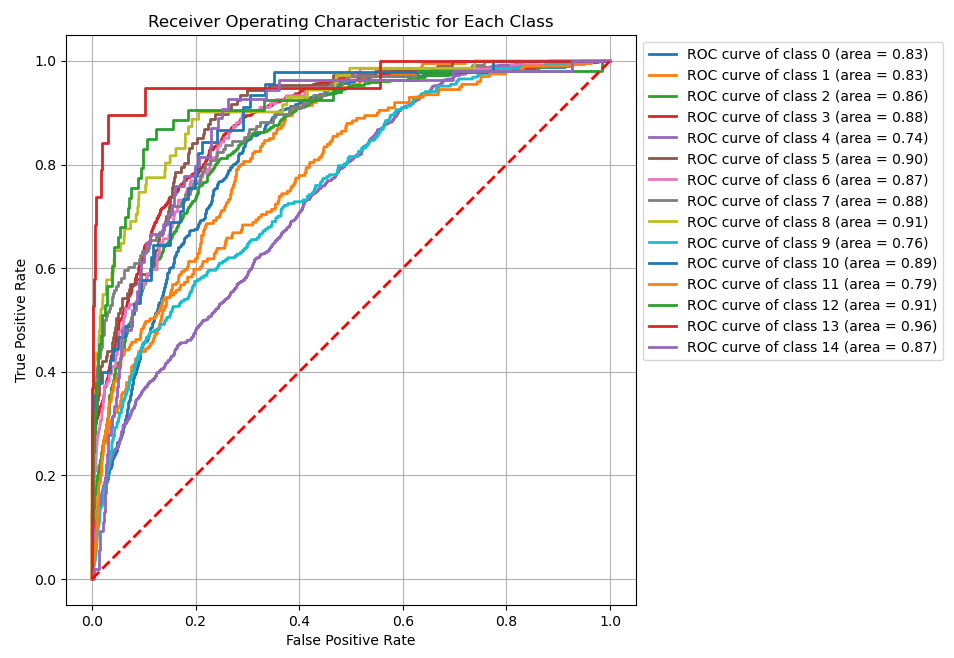

Supplement: qzaf093_Supplementary_Data [file qzaf093_supplementary_data.zip › Figure S2.png]

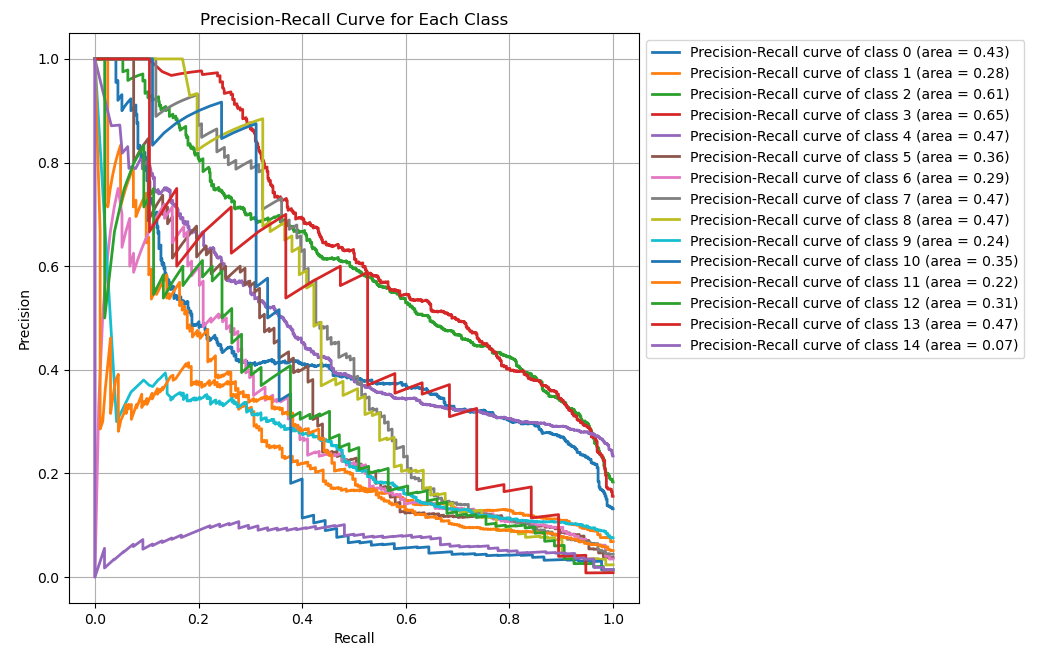

Supplement: qzaf093_Supplementary_Data [file qzaf093_supplementary_data.zip › Figure S3.png]

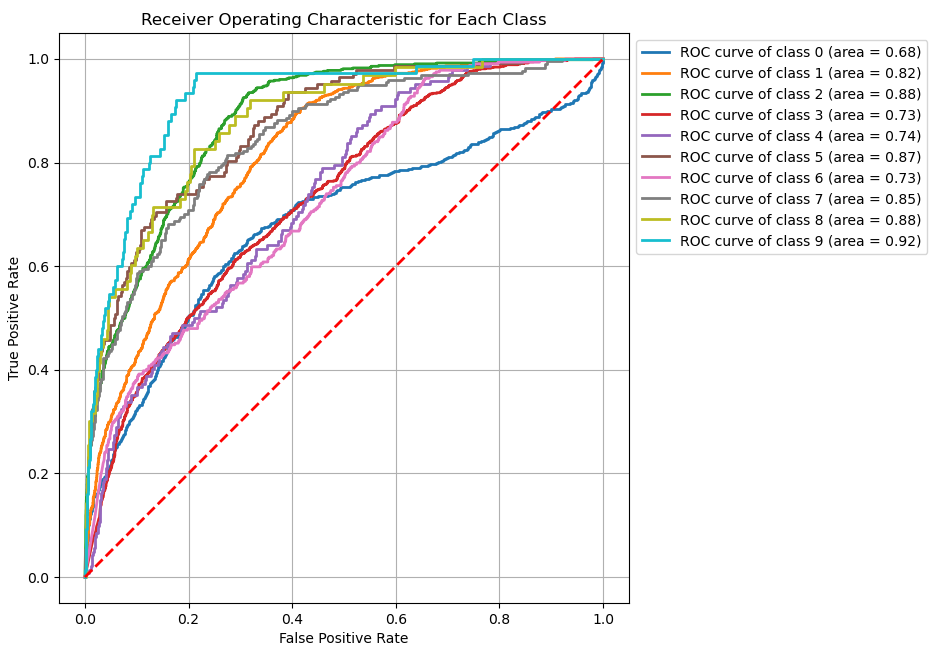

Supplement: qzaf093_Supplementary_Data [file qzaf093_supplementary_data.zip › Figure S4.png]

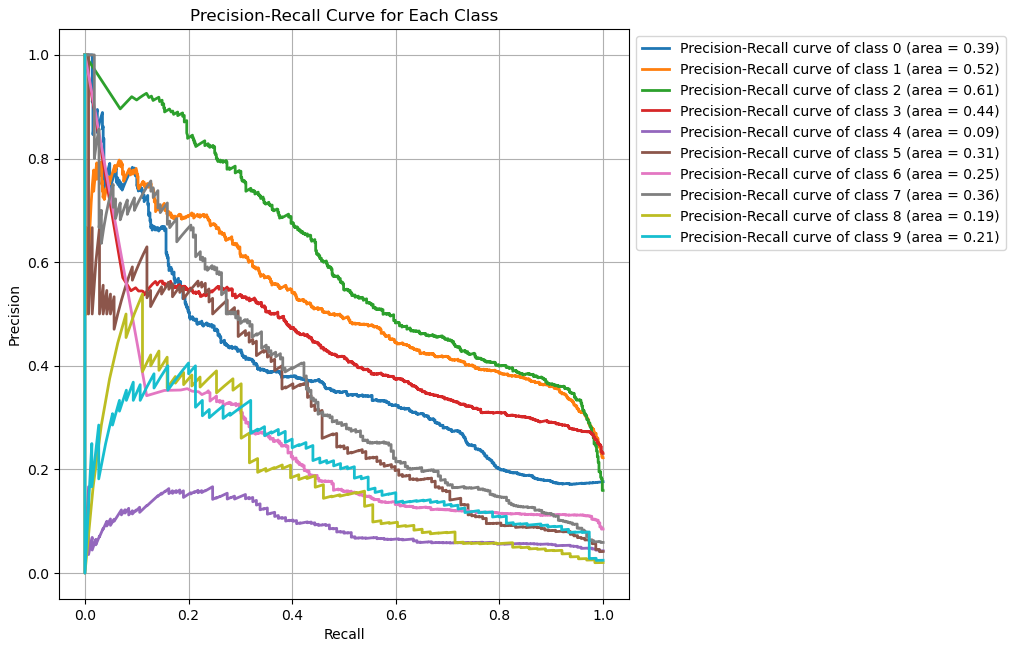

Supplement: qzaf093_Supplementary_Data [file qzaf093_supplementary_data.zip › Figure S5.png]
